# The Repetition of the Homœopathic Remedy

**Published:** 1889-03

**Authors:** Samuel Hahnemann

**Affiliations:** Kothen


### HOMŒOPATHIC PHYSICIAN,

A MONTHLY JOURNAL OF

HOMŒOPATHIC MATERIA MEDICA AND CLINICAL MEDICINE.

"If our school ever give up the strict inductive method of Hahnemann, we are lost, and deserve only to be mentioned as a caricature in the history of medicine."—CONSTANTINE HERING.

Vol. IX. MARCH, 1889. No. 3.

### THE REPETITION OF THE HOMŒOPATHIC REMEDY.

(Translated from the German by F. H. Lutze, M. D., Cheshire, N. Y.)

In the previous editions of the *Organon* I recommended the necessity of allowing the single dose of the well-selected homœopathic remedy, given at once, to exhaust its action before a new one be given or the previous one repeated. This doctrine originated in the positive experience that a larger dose of the remedy, though well chosen (as lately again proposed like a retrograde movement), or, which is the same, that several small doses repeated at short intervals, hardly ever can produce the greatest possible good in the cure of any but especially chronic disease. For by such a procedure the life-force does not accommodate itself quietly from the disturbance of the natural disease into a change to a similar drug disease, but revolts from a large dose, or even several smaller ones quickly and frequently repeated, even of a well-selected homœopathic remedy, so that the reaction is in most cases nothing less than curative, produces, on the contrary, more harm than good. Since then no procedure more helpful than the one previously taught by me could be discovered, the humane rule: "*Si non juvat, modo ne noceat,*" commanded the homœopathic physician, who would make the greatest good to mankind his highest aim, to give for disease in general only one single dose of the carefully-selected remedy at a time, and that the smallest, and to allow this to act till its action is exhausted. I say the smallest, for it is and ever will be a homœopathic law of cure, which cannot be refuted by any experience in the world, that the smallest dose of a high potency (X) of the correctly selected remedy, is its best dose in chronic as well as in acute diseases, a truth, the inestimable property of pure Homœopathy, which will separate by a chasm, across which no one can look, the false healing arts from pure Homœopathy, so long as the allopathy (and likewise the new mixed sect composed of both allopathic and homœopathic experience) continue, like cancer, to undermine the lives of the sick and to destroy them with larger or very large doses of medicine.

On the other hand, however, practice teaches us that a single one of these smallest doses will, perhaps, in some very light cases of disease, especially in small children and delicate and very susceptible adults, be sufficient to do all that medicine so far can do; that, however, in other cases, indeed in most cases of continued as well as too far progressed, often by previous drugging complicated, as also in grave acute diseases, plainly such a minimum dose of a remedy, even in our highly dynamized potency, is insufficient to produce all the curative effects which we can possibly expect to be produced by this same remedy; for here it is undoubtedly necessary to give several of such small doses, so that the life-force may be pathogenetically changed to that degree, and the curative reaction so increased that it may be enabled to eradicate all of the original disease, which the well-selected homœopathic remedy has the power to eradicate and completely obliterate the same through its counteraction. The best-selected remedy in so small a dose, given once only, would give in such cases some relief, but not by far enough.

To repeat the same dose of the same remedy soon again and again, the homœopathic physician would not dare to, since he has learned from careful observation that he did not derive any benefit therefrom, but that it resulted, on the contrary, oftenest in a great deal of positive injury. He generally noticed an aggravation, when, after giving to-day even the smallest dose of the suitable remedy, he repeated the same to-morrow and again the day thereafter.

Now, to benefit his patient more than could be done by administering a single small dose of the remedy, of whose most careful choice and homœopathicity he was fully convinced, the idea occurred to him naturally, since on account of above reasons it should be but one dose, to increase the dose, and, instead of a single one of the smallest globules moistened with the remedy in the highest potency, to give, perhaps, six, seven, or eight at once, or even one-half drop or a whole one. The result of this, however, was almost invariably (or without exception) less favorable than it should have been, often even unfavorable, or indeed, very injurious, an injury which in a patient so treated is very difficult to make good again.

To give a large dose of the remedy in a lower potency is in such a case really a no better expedient.

An increase in the strength of the single dose of the homœopathic remedy, until it produce the desired and necessary degree of the pathogenetic stimulation of life-force toward a sufficiently curative reaction, does not fulfill the desired purpose by any means, as experience also teaches us. The life-force is in this way too suddenly and too strongly attacked, so that she has no time for an even and gradual curative reaction to accommodate itself to this change, wherefore she will try to reject the excess of the remedy assaulting her like an enemy, by vomiting, diarrhœa, fever, sweat, etc., and in this way she destroys and frustrates the aim of the inconsiderate physician for the greater part or entirely. Very little or no good for the cure of the patient is thereby accomplished ; on the contrary, the patient is thereby visibly enfeebled, and we dare not think of it even for a long time thereafter, to give the patient even the smallest dose of the same remedy, lest it might prove very detrimental to him.

Likewise, a number of the smallest doses given for the same purpose in rapid succession accumulate in the organism to an over-large dose, with similar evil consequences, with few and rare exceptions; the life-force is in such cases oppressed and overwhelmed, and unable in the interval between the two, though small, doses to recuperate; incapable to react in the direction toward health, she is compelled to continue passively and involuntarily the too powerful drug-disease with which she has been overburdened, similarly, as we daily perceive, from the allopathic abuse of large, heaping doses of one and the same remedy, to the lasting injury of the patient.

Now, to attain our end with more certainty than heretofore, avoid the wrong methods indicated above, and give the chosen remedy in such a way that it may, without injury to the patient, reach its highest point of activity ; and to produce in a given case of disease all the good possible, which it is able to produce, I have of late adopted a peculiar method.

I recognized, that, to arrive at the proper middle road, the nature of the different remedies, as well as the peculiar idiosyncrasy of the patient, and the severity of the disease would have to be considered ; and, to give an example of the use of *Sulphur* in chronic (psoric) diseases, that the smallest dose of the same

(*Tinct. Sulph. X°*), even in strongly constituted persons and developed psora could rarely be oftener repeated with benefit than every seven days, an interval of time which ought to be prolonged the more when debilitated and excitable people are to be treated, for then it is better to give such a dose only every nine, twelve, or fourteen days, which should be continued to be repeated only so long till the remedy ceases to be useful. Then we will find (to continue with *Sulph.* as the example) that it requires in psoric diseases rarely less than four, but often six, eight, and even ten such doses in like periods one after the other (*Tinct. Sulph. X°*) to completely cure that much of the chronic disease, which *Sulphur* at best is able to cure, provided that no allopathic abuse of *Sulphur* has preceded this. Thus can even a newly formed (primary) itch eruption, in persons not too delicate, even if the whole body should be covered completely, be cured inside of ten to twelve weeks by a dose of *Tinct. Sulph. X°*, given every seventh day (that is, then with ten to twelve globules), so that it is hardly ever necessary to give a couple of doses of *Carbo veg. X°* (also one every week) as an auxiliary remedy, without the least external treatment, except clean underclothing and proper hygiene and diet.

If, in other chronic diseases, after due consideration, eight, nine, or ten doses of *Tinct. Sulph. X°* are deemed necessary, yet in such cases it is to be preferred, instead of giving these in an uninterrupted succession, to interpolate after every three doses a dose of some other perfectly homœopathic acting remedy and to allow this also to act eight, nine, twelve, or fourteen days before beginning again to give a series of three successive doses of *Sulphur*. This intercurrent remedy is best such a one as would be thought prudent to give a couple of times in succession with intervals of eight to fourteen days, after the treatment with *Sulphur* is concluded.

Not very rarely, however, the life-force rebels rather than to allow several doses of *Sulphur*, given at intervals as stated above, quietly to act upon itself, though the same might be ever so useful for the chronic evil, and shows this antagonism by producing, during the treatment of the invalid, a few though mild *Sulphur symptoms*. Then it is sometimes advisable to give a dose of *Nux-vom. X°*, and allow this to act for eight to twelve days, so that nature may be induced to allow the *Sulphur* in continued doses to act again quietly and with the greatest possible benefit. In suitable cases, *Puls. X°* may be preferred.

The life-force shows itself, however, most averse to allow *Sulphur*, though decidedly indicated, to act upon it, shows even visible aggravations of the chronic disease, even after the smallest dose of *Sulphur*, yes, even after the smelling of a globule no larger than a mustard-seed, moistened with *Tinct. Sulph. X°*, if *Sulphur* has been, even several years previously, in large allopathic doses abused.

This is, among many others, the most deplorable condition which frustrates almost entirely the best medical treatment of a chronic disease, and would cause us to deplore the common maltreatment of chronic diseases by the old school if there were no remedy for this.

In such cases it is only necessary to allow the patient to inhale only once the vapor of a globule the size of a mustard-seed moistened with *Mercur. Metall. X°*, and to allow this inhalation to act for nine days. Thus the life-force is induced to allow the *Sulphur*, at least by smelling on *Tinct. Sulph. X°*, to exert again a beneficial influence upon itself, a discovery for which we have to thank *Dr. Grieszlich, of Carlsruhe.*

Of other anti-psoric remedies (with the exception of *Phosph. X°*), it is necessary not to give so many doses with similar intervals (of *Sepia* and *Silicea*, when homœopathically indicated at longer intervals and without intercurrents) to see our expectations fulfilled, and cure with the indicated remedy all that possibly can be cured in a given case. *Hep. sulp. calc. X* cannot be given internally or by inhalation with shorter intervals than every fourteen to fifteen days.

It is obviously necessary that the physician who would undertake such a repetition of doses must be beforehand convinced of the correct homœopathic choice of the remedy.

In acute disease the repetition of the suitably chosen remedy must be regulated according to the more or less rapid course of the disease to be overcome so that it is to be repeated if necessary after twenty-four, twelve, eight, four hours, or even less in case the remedy causes improvement without causing new difficulties, but not quickly enough considering the rapid and dangerous progress of the acute disease, so that in the most rapidly death-producing disease of which we know—in *cholera* —at the outbreak of the same we must give every five minutes one to two drops of a weak solution of *Camphor* to render quick and sure assistance, but by more developed *cholera*, also doses of *Cuprum*, *Veratrum*, *Phosphorus*, etc. (X°), after every two or three hours, as perhaps, also *Arsenic*, *Carbo-veg.* in similar short intervals.

In the treatment of the so-called nervous fever (typhus) and other continuous fevers, the same rule as above must be observed regarding the repetition of the curative-acting remedy in the smallest dose.

In syphilitic diseases of the pure type, I generally found a single dose of *Hydrargyrum metallicum* (*Merc.-viv.*) X° sufficient, yet not rarely were two or three such doses necessary to be given in intervals of six to eight days if there was but the least complication with psora visible.

But especially in the form of vapor by smelling and inhaling the continuously evaporating remedial vapors from one with the dilution of a high potency, moistened globule, lying dry in a vial, do the homœopathic remedies act with the greatest certainty and power. The homœopathic physician will let the patient hold the mouth of the uncorked vial, first in one nostril to inhale with the act of inspiring the air out of this, and then, perhaps, if the dose is to be stronger to inhale likewise through the other nostril more or less strongly, as he may order the dose, and then put it carefully corked again in his pocket-case, so that no abuse can be committed with it, *and so if he does not choose he will require no apothecary any more for his cures.* A globule, ten to twenty of which weigh one grain, moistened with the 30th potency and then dried, retains its full power for this purpose undiminished for at least eighteen to twenty years (so far goes my own experience), even though the vial should have been opened in the meantime a thousand times, provided it is only protected from heat and sunlight. Even should both nostrils be obstructed by chronic catarrh or polypi, the patient may inhale by the mouth, holding the mouth of the vial between his lips. In small children you may hold it during sleep close to one and then the other nostril, and can be positive of the result. This inhaling of the vapor of the remedy affects the nerves in the walls of the roomy cavities through which it passes without hindrance, and so affects the life-force curatively in the mildest and yet most powerful manner, preferably to any other way of administering the remedy in substance by the mouth. All that can be cured by Homœopathy (and what diseases, except such as require actual manual surgical interference, can she not cure?), the most chronic, not by allopathy altogether spoiled and corrupted, as well as acute diseases will be cured by this inhaling in the most safe and sure manner. Among the many sick who sought for the past year and more mine and my assistants' aid, I can call to mind hardly one in a hundred whose chronic or acute disease we did not treat with the desired success by means of such inhalations. In the last half this year, however, I have arrived at the conviction (which no one could have made me believe before) that the power of the remedy by inhalation in this way is exerted upon the sick in the same degree of force, yet quieter and fully as long as the dose taken by the mouth, and that, therefore, the time to repeat the inhalation should not be ordered shorter than when taking the material dose by the mouth. SAMUEL HAHNEMANN.

KOTHEN, May, 1833.

